# Movement- and Posture-based Measures of Sedentary Patterns and Associations with Metabolic Syndrome in Hispanic/Latino and non-Hispanic Adults

**DOI:** 10.1007/s40615-024-02114-w

**Published:** 2024-08-12

**Authors:** Marta M. Jankowska, Calvin P. Tribby, Paul R. Hibbing, Jordan A. Carlson, Mikael Anne Greenwood-Hickman, Dorothy D. Sears, Andrea Z. LaCroix, Loki Natarajan

**Affiliations:** 1 Department of Population Sciences, Beckman Research Institute, City of Hope, Duarte, CA USA; 2 Department of Kinesiology and Nutrition, University of Illinois Chicago, Chicago, IL USA; 3 Center for Children’s Healthy Lifestyles & Nutrition, Children’s Mercy Kansas City, Kansas City, MO USA; 4 Department of Pediatrics, University of Missouri Kansas City, Kansas City, MO USA; 5 Kaiser Permanente Washington Health Research Institute, Seattle, WA USA; 6 UC San Diego Moores Cancer Center, San Diego, CA USA; 7 College of Health Solutions, Arizona State University, Phoenix, AZ USA; 8 Department of Family Medicine, University of California San Diego, La Jolla, San Diego, CA USA; 9 Herbert Wertheim School of Public Health and Human Longevity Science, University of California San Diego, La Jolla, San Diego, CA USA

**Keywords:** Health disparities, Diabetes, Behavior modification, Stratified analysis, Energy expenditure, Machine learning

## Abstract

**Background:**

Sedentary behavior has been identified as a significant risk factor for Metabolic Syndrome (MetS). However, it is unclear if the sedentary pattern measurement approach (posture vs. movement) impacts observed associations or if associations differ for Hispanic/Latino communities, who have higher risk of MetS.

**Methods:**

Participants from the Community of Mine (CoM) study (N = 602) wore hip-based accelerometers for 14 days and completed MetS-associated biomarker assessment (triglycerides, blood pressure, fasting glucose, HDL cholesterol, waist circumference). Sedentary patterns were classified using both cutpoints (movement-based) and the Convolutional Neural Network Hip Accelerometer Posture (CHAP) algorithm (posture-based). We used logistic regression to estimate associations between MetS with sedentary patterns overall and stratified by Hispanic/Latino ethnicity.

**Results:**

CHAP and cutpoint sedentary patterns were consistently associated with MetS. When controlling for total sedentary time and moderate to vigorous physical activity, only CHAP-measured median sedentary bout duration (OR = 1.15, CI: 1.04, 1.28) was significant. In stratified analysis, CHAP-measured median bout duration and time spent in sedentary bouts ≥ 30 min were each associated with increased odds of MetS, but the respective associations were stronger for Hispanic/Latino ethnicity (OR = 1.71 and 1.48; CI = 1.28–2.31 and 1.12–1.98) than for non-Hispanic/Latino ethnicity (OR = 1.43 and 1.40; CI = 1.10–1.87 and 1.06–1.87).

**Conclusions:**

The way sedentary patterns are measured can impact the strength and precision of associations with MetS. These differences may be larger in Hispanic/Latino ethnic groups and warrants further research to inform sedentary behavioral interventions in these populations.

**Supplementary Information:**

The online version contains supplementary material available at 10.1007/s40615-024-02114-w.

## Introduction

Metabolic syndrome (MetS) is a group of conditions including abdominal obesity, high blood pressure, high blood sugar levels, high blood triglycerides, and low HDL cholesterol, which together raise an individual’s risk for heart disease, diabetes, and several cancers [1]. Sedentary behavior, both self-reported and device-measured, has been identified as a significant risk factor for MetS [2–7]. Sedentary behavior is defined as all waking behaviors while in a sitting or lying position with energy expenditure ≤ 1.5 metabolic equivalents [8]. Sedentary behavior can be measured as total time spent being sedentary, or as sedentary patterns that are regular discernible sequences of sedentary behavior accumulation such as bouts or diversity of bout length [9]. Some studies investigating sedentary patterns have found that increased breaks in sedentary time and shorter sedentary bouts have beneficial effects on MetS components and related outcomes [10–13]. However, these studies have used measures that capture low movement (e.g., low accelerometer counts) but not the postural sitting or lying component key to the sedentary behavioral definition. Recent studies have shown that movement-based measures (e.g., cutpoints) show different rates of sedentary patterns than posture-based measures (e.g., activPAL) [14–16].

Research on sedentary patterns using posture-based measures and MetS or related health outcomes is limited and has produced mixed results [6, 17]. Reported study differences may underscore the sensitivity of posture-based measures to sedentary behaviors in a particular cohort and emphasize the need for a larger body of evidence examining the relationship between posture-based sedentary behaviors and MetS. This may be particularly true for ethnic minorities who experience an increased risk of MetS, and for whom posture-based interventions may help to reduce observed disparities.

Hispanics/Latinos in the US are at increased risk of MetS and suffer from increased rates of diabetes compared to non-Hispanic whites [18–21]. While observational studies suggest less sitting time among Hispanic/Latino adults compared to non-Hispanic whites, there is not a well-established body of literature on the relationship between MetS and sedentary time or patterns in Hispanic/Latino populations [22–24]. Furthermore, reduction of sedentary time may attenuate genetic associations with obesity in this population [25]. There are few assessments of sedentary patterns in the adult Hispanic/Latino population, all of which utilize movement-based measures. Only three studies currently relate sedentary patterns to metabolic outcomes in Hispanics/Latinos, finding significant and deleterious associations of prolonged and uninterrupted sedentary bouts with glucose biomarkers, obesity, and cardiometabolic biomarkers. [26–28]

Here we build upon previous literature by comparing associations of movement- and posture-based sedentary patterns to MetS in an ethnically diverse cohort. We draw on the Convolutional Neural Network Hip Accelerometer Posture (CHAP) algorithm; a deep neural network algorithm developed to classify sitting/lying versus non-sedentary behavioral postures [29, 30]. We add to limited research on sedentary pattern effects on MetS in minorities by assessing if associations between sedentary patterns and MetS are different for Hispanic/Latino and non-Hispanic individuals.

## Methods

### Participants

The Community of Mine (CoM) study enrolled 602 participants aged 35–80 years living in San Diego County from 2014–2017. The study collected behavioral, clinical, and biomarker outcomes related to cancer risk with the main study aim of advancing cancer risk exposure assessment; full details have been published elsewhere [31]. The cross-sectional study recruited adults able to walk without human assistance and willing to wear study sensors for two weeks, with a goal of oversampling participants identifying as Hispanic/Latino. Ethics approval was obtained from the University of California San Diego Institutional Review Board (protocol #140,510).

### Metabolic Syndrome and Covariates

Participants attended a clinic visit where blood pressure, height, and weight were recorded, and fasting blood samples were collected. Metabolic syndrome was defined using diagnostic criteria of the American Heart Association and National Heart, Lung, and Blood Institute, updated from the NCEP ATP III definition [32]. The presence of MetS (yes/no) was indicated if ≥ 3 of the following conditions were met: 1) elevated waist circumference (≥ 102 cm in males, ≥ 88 cm in females); 2) elevated triglycerides (≥ 150 mg/dL); 3) reduced HDL cholesterol (HDL-C; < 40 mg/dL in males, < 50 mg/dL in females); 4) elevated blood pressure (≥ 130 mmHg systolic or ≥ 85 mmHg diastolic); or 5) elevated fasting glucose (≥ 100 mg/dL).

Demographic covariates were assessed via survey and included age (years), sex (female or male), education (high school or less; associates/bachelor’s degree; graduate degree or higher), and ethnicity (Hispanic/Latino or not). Medications taken by participants were noted during their clinic visit and categorized by their clinical indication (summary in Supplement Table 1). A covariate was included for all participants based on if they were taking medications for the treatment of MetS components (yes/no) including elevated triglycerides, reduced HDL-C, elevated glucose, or hypertension.

### Sedentary Behavior Measurement and Definitions

Participants were asked to wear an ActiGraph GT3X + accelerometer (ActiGraph LLC, Pensacola, FL, USA) on the right hip for 14 days. Non-wear time was identified using the validated Choi algorithm for vector magnitude signal [32]. Valid wear days were defined as having ≥ 10 h of wear time and participants were required to have at least 2 valid wear days. Movement-based measures of total sedentary time and sedentary patterns were derived using count per minute (cpm) cutpoint thresholds consistent with standard practice in accelerometry [33]. A cutpoint threshold of < 100 cpm on the vertical axis was used to classify 1-min epochs as sedentary time. Moderate to vigorous physical activity (MVPA) was also derived using the cutpoint method and was defined using the Troiano cutpoint of ≥ 2020 cpm. [34] Posture-based measures of total sedentary time and sedentary patterns were calculated using the CHAP-adult algorithm, which has been described in detail elsewhere [29, 30, 35], and can be found online [36]. Briefly, CHAP calculates sedentary behavior by defining bouts of sitting and/or lying postures as well as posture transitions from sitting/lying to standing using hip-worn triaxial accelerometer data. It has a mean balanced accuracy of 93% for distinguishing sitting and non-sitting as validated against activPAL posture measures. Here, CHAP-adult was run on the 10-Hz raw triaxial ActiGraph data, and the output was given in 10-s epochs covering the same overall time span as the 60-s epoch cutpoint output. Non-wear time and valid wear days were matched between methods by cross-referencing the corresponding 60-s epoch data for each 10-s CHAP epoch.

For both the cutpoint (movement-based) and CHAP (posture-based) methods, sedentary time and sedentary patterns were analyzed using the *sb_summary_bouts* function in the *PBpatterns* R package (version 0.1.0.9000), which outputs a variety of summary measures related to sedentary behaviors [37]. The full list of measures, descriptions, and calculation is in Supplement Table 2. Bouts were defined as periods of sedentary time lasting ≥ 1 min and a break in sedentary time was defined as any time a sedentary minute was followed by a non-sedentary minute (no allowance for interruptions, i.e., no tolerance). Sedentary pattern variables included time in bouts lasting ≥ 30 min, the median duration of all sedentary bouts, and the number of daily breaks in sedentary time (Supplement Table 2). Percentage of wear time spent sedentary (a standardized indicator of total sedentary time) was calculated by dividing total sedentary time by total wear time.

### Statistical Analysis

Descriptive statistics were calculated for the full sample and stratified by MetS (yes/no). Additional descriptive statistics were calculated for the sedentary time and pattern measures by MetS and Hispanic/Latino ethnicity. Pearson correlations within cutpoint and CHAP measures were calculated to aid in selection of measures, focusing on those least correlated with each other to reduce redundancy in modeling. Paired *t* tests assessed differences between cutpoint and CHAP measures of sedentary time and patterns for the full sample, by MetS, and by Hispanics/Latino ethnicity. Logistic regression models assessed cutpoint and CHAP sedentary time and pattern associations with MetS, including the covariates of sex, age, education, ethnicity, MetS-related medication use, and device wear time. Two models were assessed: the base model with covariates only and the fully adjusted model which additionally controlled for MVPA and percent of total time sedentary. Interaction analysis was conducted for both models to test interaction between each sedentary pattern*Hispanic/Latino ethnicity, with likelihood ratio test performed to assess significant model fit improvement between the original and interaction model. Finally, models were stratified by Hispanic/Latino ethnicity. Results are reported as standardized odds ratios with confidence intervals, with significance set at p < 0.05 (two-tailed). All statistical analyses were conducted in R v.4.1.2 (The R Foundation for Statistical Computing, Vienna, Austria).

## Results

### Study Sample

From the 602 participants enrolled, a final sample of 579 were included in this study. We excluded participants that did not have covariate or outcome data (n = 18) or did not have sufficient valid wear accelerometer data (n = 5). Participants wore accelerometers for an average of 12.5 valid days (min 2, max 25, std 2.6), with an average of 14.4 h of daily valid wear time (min 11.6, max 20.3, std 1.3). In total, 26.4% of participants met three or more conditions for classification as having Metabolic Syndrome. More Hispanic/Latino participants had MetS (14.7%) than non-Hispanic participants (11.7%). Those with MetS averaged about 10 fewer minutes of MVPA per day compared to participants without MetS (Table 1).

**Table 1:** Descriptive statistics for the sample (n = 579) and stratified by participants with MetS (n = 153) and without (n = 426)

Variable	Total Sample n = 579		With Metabolic Syndrome n = 153		Without Metabolic Syndrome n = 426	
	Mean or n	SD or %	Mean or n	SD or %	Mean or n	SD or %
Age (years)	58.8	11.0	60.1	10.5	58.4	11.2
Sex						
Female	323	55.8	85	55.6	238	55.9
Male	256	44.2	68	44.4	188	44.1
Ethnicity						
Hispanic/Latino	242	41.8	85	55.6	157	36.9
Non-Hispanic	337	58.2	68	44.4	269	63.1
Education						
High school or less	94	16.2	36	23.5	58	13.6
Associates/Bachelor’s degree	323	55.8	89	58.2	234	54.9
Graduate degree or higher	162	28.0	28	18.3	134	31.5
MetS Medications	230	39.7	85	55.6	145	34.0
Wear time (hours per day)	14.4	1.3	14.3	1.3	14.4	1.2
MVPA Troiano (min per day)	27.2	24.1	19.6	18.8	30.0	25.2
MetS Components*						
Elevated waist circumference	275	47.5	127	83.0	148	34.7
Elevated triglycerides	118	20.4	83	54.2	35	8.2
Reduced HDL cholesterol	119	20.6	79	51.6	40	9.4
Elevated blood pressure	243	42.0	111	72.5	132	31.0
Elevated fasting glucose	275	47.5	129	84.3	146	34.3

^*^Elevated waist circumference (≥ 102 cm in males, ≥ 88 cm in females); Elevated triglycerides (≥ 150 mg/dL); Reduced HDL cholesterol (HDL-C; < 40 mg/dL in males, < 50 mg/dL in females); Elevated blood pressure (≥ 130 mmHg systolic or ≥ 85 mmHg diastolic); Elevated fasting glucose (≥ 100 mg/dL)

### Comparing cutpoint and CHAP measures of sedentary behavior

Sedentary time and pattern correlations for all cutpoint and CHAP measures are in Supplement Figs. 1 and 2, respectively. Several variables were highly correlated for both cutpoint and CHAP, including mean sedentary bout duration, sedentary bout frequency, and sedentary hours per day. Thus, these variables were excluded from further analysis. Lower correlations were observed for percent total sedentary time (0.82), mean sedentary breaks per day (0.54), median sedentary bout duration (0.53), and hours in bouts ≥ 30 min (0.82), which were retained for further analysis. All correlations were significant at p < 0.001.

For the total sample, the cutpoint measure of mean percent total sedentary time (61.1%, SD = 10.4%) and CHAP measured time (60.7%, SD = 12.2%) were not significantly different, nor when stratified by MetS (Table 2). When stratified by Hispanic/Latino ethnicity, cutpoint mean percent total sedentary time, 58.2% (SD = 11.0%) was significantly higher than CHAP measured time, 57.0% (SD = 12.5%; p = 0.009) for Hispanic/Latinos (Table 2). While estimates of total sedentary time were largely similar between cutpoint and CHAP, all of the sedentary patterns were significantly different. For example, cutpoint estimates of mean sedentary breaks per day, 83.0 (SD = 15.7) was higher than CHAP estimates, 44.3 (SD = 11.9; p < 0.001) for the total sample and this relationship was similar across MetS and Hispanic/Latino stratifications (Table 2).

**Table 2:** Cutpoint and CHAP sedentary measures (means and standard deviation) for the total sample and stratified by MetS and ethnicity. Paired *t* test p values comparing cutpoint to CHAP measures in each sample (total, MetS, without MetS, Hispanic/Latino, non-Hispanic)

Sedentary Measures mean (standard deviation)	Total n = 579	With MetS n = 153	Without MetS n = 426	Hispanic/ Latino n = 242	Non-Hispanic n = 337
Cutpoint Sedentary Measures					
Percent total sed time	61.1 (10.4)	63.3 (11.1)	60.4 (10.1)	58.2 (11.0)*	63.3 (9.5)
Mean sed breaks / day	83.0 (15.7)**	80.8 (16.7)**	83.8 (15.3)**	85.8 (15.4)**	81.0 (15.6)**
Median sed bout duration (min)	2.6 (0.7)**	2.7 (0.8)**	2.5 (0.7)**	2.4 (0.6)**	2.7 (0.8)**
Hours in bouts ≥ 30 min	2.8 (1.6)**	3.0 (1.7)**	2.7 (1.5)**	2.3 (1.4)**	3.1 (1.6)**
CHAP Sedentary Measures					
Percent total sed time	60.7 (12.2)	63.3 (12.3)	59.8 (12.0)	57.0 (12.5)	63.3 (11.2)
Mean sed breaks / day	44.3 (11.9)	42.2 (11.5)	45.1 (11.9)	44.6 (11.6)	44.1 (12.1)
Median sed bout duration (min)	5.0 (2.2)	5.6 (2.3)	4.8 (2.1)	4.5 (1.8)	5.4 (2.4)
Hours in bouts ≥ 30 min	4.5 (1.9)	4.9 (2.0)	4.4 (1.9)	4.0 (1.8)	4.9 (2.0)

p < 0.01*, **p < 0.001

### Associations of Sedentary Patterns with Total Sample MetS

Model 1 results show significant associations of similar effect magnitude (OR = 0.78) between both cutpoint and CHAP measures of mean breaks per day and MetS, and stronger associations for CHAP measures of median bout duration and hours in bouts ≥ 30 min compared to cutpoint measures (Table 3). A one standard deviation increase in cutpoint measured median sedentary bout duration was associated with 1.36 times the odds of MetS (p = 0.002), whereas a one standard deviation increase in CHAP measured median sedentary bout duration was associated with 1.56 times the odds of MetS (p < 0.0001).

**Table 3:** Logistic regression results for cutpoint and CHAP measured sedentary pattern associations (odds ratios, 95% confidence intervals) with the outcome of MetS. Model 1 controls for covariates and model 2 additionally controls for both MVPA and total sedentary time

Sedentary measures N = 579	Model 1: Covariates	p	Model 2: Covariates + % total sedentary + MVPA	p
Mean breaks per day				
Cutpoint	0.78 (0.61, 0.99)	0.04	0.99 (0.97, 1.00)	0.14
CHAP	0.78 (0.62, 0.96)	0.02	0.98 (0.96, 1.00)	0.06
Median bout duration				
Cutpoint	1.36 (1.12, 1.66)	0.002	1.28 (0.86, 1.89)	0.22
CHAP	1.56 (1.28, 1.91)	< 0.0001	1.15 (1.04, 1.28)	0.008
Hours in bouts ≥ 30 min				
Cutpoint	1.38 (1.12, 1.71)	0.003	1.04 (0.83, 1.30)	0.72
CHAP	1.47 (1.20, 1.80)	0.0002	1.02 (0.84, 1.24)	0.82

Covariates: age, sex, Hispanic/Latino, education, any MetS medication, daily wear time

Model 2 controlled for both MVPA and percent of total time spent sedentary (Table 3). The one significant sedentary pattern was the CHAP median bout duration (OR = 1.15, 95% CI = 1.04, 1.28; p = 0.008).

### Associations of Sedentary Patterns with MetS Stratified by Hispanic/Latino Ethnicity

Results for interaction analysis are found in Supplement Table 3. Interaction terms (sedentary pattern*Hispanic/Latino) are not significant for any model, and all likelihood ratio test results comparing each model and model b version (with the interaction term) are null. While the interactions were not significant, there were still qualitative differences in MetS odds ratios by Hispanic/Latino for sedentary patterns. For example, a one standard deviation increase in CHAP median bouts per day for Hispanic/Latinos was associated with 1.71 times the odds of MetS (p = 0.0004), whereas for non-Hispanic/Latinos the odds were less, 1.43 (p = 0.008) (Table 4). In general, CHAP sedentary pattern measures are more precise and more consistently associated with MetS for both Hispanics/Latinos and non-Hispanics compared to cutpoint measures.

**Table 4:** Logistic regression results, stratified by Hispanic/Latino ethnicity, for cutpoint (left) and CHAP (right) sedentary pattern associations (standardized odds ratios with confidence intervals) with the outcomes of Metabolic Syndrome and constituent items with covariates (Model 1)

	Cutpoint sedentary patterns				CHAP sedentary patterns			
Sedentary measures	Hispanic/Latino	p	Non-Hispanic	p	Hispanic/Latino	p	Non-Hispanic	p
Mean breaks per day	0.76 (0.53, 1.09)	0.14	0.77 (0.55, 1.07)	0.12	0.74 (0.54, 1.00)	0.06	0.81 (1.58, 1.10)	0.20
Median bout duration	1.34 (1.02, 1.78)	0.04	1.34 (1.02, 1.77)	0.04	1.71 (1.28, 2.31)	0.0004	1.43 (1.10, 1.87)	0.008
Hours in bouts ≥ 30 min	1.30 (0.98, 1.75)	0.07	1.42 (1.05, 1.92)	0.02	1.48 (1.12, 1.98)	0.006	1.40 (1.06, 1.87)	0.02

Covariates: age, sex, education, any MetS medication, daily wear time

## Discussion

This study compared associations between movement-based cutpoint and posture-based CHAP sedentary measures with Metabolic Syndrome in a sample of 579 adults, with stratified analysis for Hispanic/Latino ethnicity. While CHAP and cutpoint measures estimate similar total amounts of sedentary time, CHAP estimated less interrupted patterning (half the number of breaks, double the length of bouts) of sedentary behavior, all of which were significantly different from cutpoint measures. One important finding is that these patterns held consistent when stratified by MetS or Hispanic/Latino, except for percent total sedentary time. In previous research in an older adult population, agreement for total sedentary time, hours in bouts ≥ 30 min, and breaks in sedentary time between methods had a Pearson correlation of 0.66, 0.78, and 0.51 respectively. [15] In a study of 1,457 people, Edwardson et al. saw a lower between-method correlation of total sedentary time (0.51) and for average number of breaks in sitting per day (0.42) [38]. Taken together, these findings build a body of evidence across age spectrums and health outcomes that while there is general agreement between movement- and posture-based measures in detecting total sedentary time and sedentary patterns, movement-based measures detect more breaks in sedentary time than posture-based measures [16]. This study adds that there may be differences in movement and postural patterns in Hispanics/Latinos that warrant further study.

Model results had expected direction of associations between both measurement methods with MetS. Both longer bout durations and hours in bouts ≥ 30 min were associated with greater odds of MetS, while increasing breaks per day lowered odds. Levels of PA are an important factor, independent of sedentary behaviors, in the study of sedentary behaviors and MetS [13, 39]. There is some debate as to the need for adjusting for PA in studies of sedentary behavior [40], however, time spent in PA and higher intensities of PA have been shown to lower odds of MetS [41–43]. Replacing sedentary time with PA was associated with greater MetS risk reduction than modifying either behavior alone [44]. Furthermore, breaking up sedentary time with PA breaks is associated with a reduction in some components of MetS [45]. To assess these two factors, model 1 controlled only for covariates and model 2 added MVPA and percent of time in sedentary. In model 2, only CHAP measured median bout duration was significant, indicating that sedentary bout duration may have a biomechanical pathway to MetS independent of total sedentary time and MVPA.

An important contribution is the stratified analysis focusing on the Hispanic/Latino participants compared to non-Hispanic participants. Hispanic/Latino participants were 1.7 times more likely to have MetS for every 1 standard deviation increase in median sedentary bout duration compared to non-Hispanics. However, Hispanic/Latino participants spent fewer hours in bouts ≥ 30 min (mean = 2.3) compared to non-Hispanic/Latino participants (mean = 3.1) and a lower percent of overall time sedentary, 58.2% compared to 63.3%, respectively. The significant finding was the discrepancy in the CHAP posture-based method’s sensitivity to significant associations in the Hispanic/Latino population compared to the cutpoint movement-based method. It is unclear if this is due to ethnic differences in posture-based sedentary behaviors, differences in the relationship between behaviors with MetS, or if perhaps the lower rates of sedentary time and more disrupted sedentary patterns in the Hispanic/Latino population necessitate more accurate measurement of each postural change. This is an area of needed further study, as our ability to accurately measure differences in behavioral patterns and their associations with MetS may be an important aspect of accounting for and intervening on metabolic health disparities in Hispanics/Latinos. In Chang et al. interaction analysis showed that association between movement-based mean sitting bout duration and fasting glucose concentration was significantly stronger among Hispanic/Latina women than non-Hispanic women [26]. Larsen et al., found that Hispanics/Latinos self-reported sitting significantly less than non-Hispanic whites, yet had more than double the odds of having diabetes after adjusting for total sitting time [24]. They concluded that differences in sedentary time likely do not contribute to ethnic disparities in type 2 diabetes, but were unable to explore sedentary patterns due to self-report measurement. Our findings indicate that sedentary patterns did have stronger associations with MetS in Hispanics/Latinos compared to non-Hispanics, and posture-based measures captured this association more consistently and precisely than movement-based measures.

As research into intervening on sedentary behaviors increases, several studies are finding replacing sitting time with walking and standing yields similar and even better improvements in glucose response, insulin resistance, triacylglycerol measures, and MetS than MVPA [45–48]. In Hispanics/Latinos, less sedentary time, but not MVPA was associated with improved cardiovascular risk factors [49]. However, many of these studies are being designed around movement-based assessment of sedentary behaviors. This study provides further evidence that not only are movement-based assessments potentially overestimating postural changes associated with sedentary behavior, but they may also not be able to precisely estimate relationships to MetS. We found this risk to be even higher in Hispanics/Latinos, where movement-based measures consistently missed associations between MetS and sedentary patterns that were significant when using posture-based measures. This is particularly relevant when designing interventions for Hispanics/Latinos. Lassalle et al. argue that targeting reduction of sedentary behaviors may be more achievable than increasing physical activity, particularly in minority populations who may enjoy fewer resources and less access to physical activity promoting environments [50]. As we move toward the development of quantitative sedentary behavior guidelines [51], it will be essential to increase our understanding of movement- and posture-based associations with MetS outcomes with a focus on minority populations.

Application of the CHAP algorithm to accelerometer data to produce posture-based measures of sedentary patterns was a key strength of the study. Use of CHAP allows for reliable, validated, objective measures of posture-based sedentary behaviors in previously collected studies using hip accelerometry. A limitation of this study was that the Hispanic/Latino population was comprised of almost all individuals from Mexico or of Mexican heritage, thus is not representative of the more diverse Hispanic/Latino population in the US. Another limitation was modeling MetS as a dichotomous variable, which may be problematic because it does not consider the risk gradient based on the individual components. Further limitations include a cross-sectional study design, where causality relationships between sedentary patterns measures and MetS cannot be determined. The positive association between increased sedentary behavior and MetS has been established by other prospective studies, affirming our findings [52, 53]. The two-week accelerometer monitoring period may not capture all usual behavior patterns of individuals, although less than two-weeks of accelerometer data has been shown to produce stable estimates of group-level means of sedentary behaviors [54, 55]. Hip-worn accelerometers may not adequately capture resistance training (e.g., weight lifting) as would wrist-worn accelerometers [56], but resistance training is likely complimentary to and does not replace aerobic activity to lower risk of MetS [57]. Finally, the role of daily accelerometer wear time in this sample (mean = 14.4 h per day) may bias the associations between sedentary time and MetS towards the null, though we attempted to account for this by adjusting models for wear time. On days with less than 14 h of wear, each hour less of wear time was associated with a lower percentage of sedentary time captured [58, 59].

## Conclusion

This study is an important contribution to a growing evidence base that not only is overall sedentary time a risk factor for MetS, but sedentary patterns play a role in MetS beyond MVPA and total sedentary time. The role of sedentary pattern measurement, using movement- or posture-based methods, impacted the strength and precision of measured associations. In stratified analyses we found that for Hispanics/Latinos posture-based measures were more sensitive to significant associations compared to movement-based measures. Further evidence investigating posture-based sedentary pattern associations with MetS in Hispanics/Latinos is needed.

## Supplementary Information

Below is the link to the electronic supplementary material.

Supplementary file1 (DOCX 345 KB)
