# Supplementary material for: Movement- and Posture-based Measures of Sedentary Patterns and Associations with Metabolic Syndrome in Hispanic/Latino and non-Hispanic Adults
Source: J Racial Ethn Health Disparities. 2024 Aug 12;12(5):3086–94. doi: 10.1007/s40615-024-02114-w (PMC12446093; doi:10.1007/s40615-024-02114-w)

**Supplement Table 1. Summary of medication counts of by category, not exclusive**

|  | Medication Category | | |
| --- | --- | --- | --- |
| Number of medications | Insulin | Hypertension | Cholesterol Lowering |
| 0 | 566 | 398 | 442 |
| 1 | 6 | 112 | 127 |
| 2 | 7 | 52 | 8 |
| 3+ | 0 | 17 | 2 |

**Supplement Table 2.** PB Pattern Library Definitions

| **Measure name (variable name)** | **Description** | **R package calculation** |
| --- | --- | --- |
| Total sedentary minutes (total_sed_min) | Total sedentary minutes | total_sed_min |
| Percent sedentary time of total time (sed_perc) | Percent of sedentary time of total wear time | total_sed_min / total_weartime_min |
| Mean sedentary breaks per day (sed_breaks_day) | Average of total sedentary bouts by number of days | n_sed_bouts / n_days |
| Median sedentary bout duration (median_bout_duration) | Median sedentary bout duration in minutes | bouts$lengths_min %>% stats::quantile(probs = 0.5) |
| Mean sedentary bout duration (mean_bout_duration) | Average sedentary duration in minutes | mean(bouts$lengths_min) |
| Bouts ≥ 30 min (hours_bouts_more_30min) | For bouts 30 minutes or longer, total time in hours | sum(ifelse(bouts$lengths_min >= 30, bouts$lengths_min, 0)) / 60 |
| Frequency of sedentary bouts (sed_bout_frequency) | Sum of all minutes in sedentary bouts by number of days | total_sed_min / (n_days * 60) |
| Bout frequency (sed_bout_frequency) | Average sedentary bouts by total minutes of wear time | n_sed_bouts / total_weartime_min * 60 |

**Supplement Table 3.** Interaction term results from logistic regressions for cut point and CHAP sedentary measure associations (standardized odds ratios with 95% confidence intervals) with the outcome of Metabolic Syndrome, interaction terms with Hispanic/Latino. Likelihood ratio test p value is provided to compare each interaction model (e.g., Model 0b) to original model without interaction term (e.g., Model 0).

| **N=579** | **Model 1b:**  **Covariates** | **LRT p** | **Model 2b:**  **Covariates**  **+ % total sed + MVPA** | **LRT p** |
| --- | --- | --- | --- | --- |
| Mean breaks per day |  |  |  |  |
| Cut point*Hispanic/Latino | 1.15 (0.94, 1.41) | 0.17 | 1.08 (0.88, 1.33) | 0.45 |
| CHAP*Hispanic/Latino | 1.02 (0.83, 1.25) | 0.87 | 0.97 (0.79, 1.19) | 0.77 |
| Median bout duration |  |  |  |  |
| Cut point*Hispanic/Latino | 1.00 (0.81, 1.22) | 0.97 | 0.97 (0.78, 1.20) | 0.76 |
| CHAP*Hispanic/Latino | 1.15 (0.93, 1.42) | 0.20 | 1.09 (0.88, 1.36) | 0.45 |
| Hours in bouts ≥ 30 mins |  |  |  |  |
| Cut point*Hispanic/Latino | 0.93 (0.76, 1.14) | 0.47 | 0.90 (0.73, 1.11) | 0.32 |
| CHAP*Hispanic/Latino | 1.05 (0.86, 1.28) | 0.63 | 1.00 (0.82, 1.23) | 0.98 |

Covariates: age, sex, Hispanic/Latino, education, any MetS medication, daily wear time


**Supplement Figure 1.** Correlations for sedentary time and patterns as measured by cutpoints.


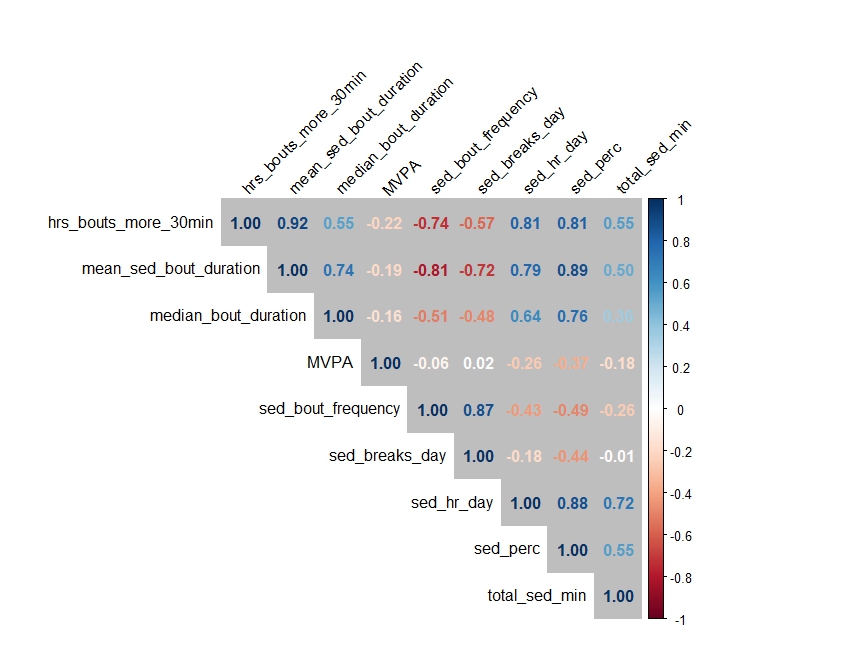


**Supplement Figure 2.** Correlations for sedentary time and patterns as measured by CHAP.


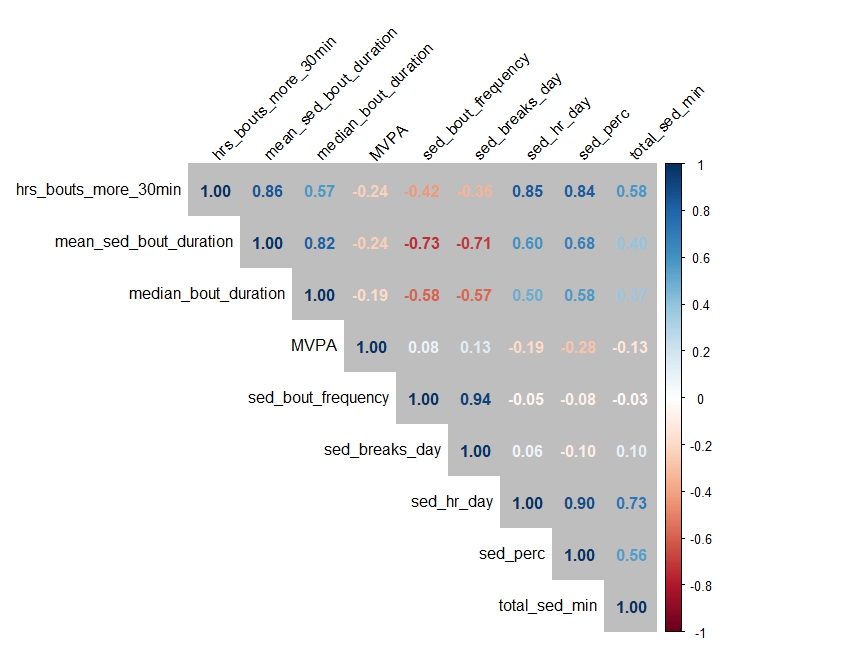

Supplement: Supplementary file 1 — Supplementary file1 (DOCX 345 KB) [file 40615_2024_2114_MOESM1_ESM.docx]
